# Impact of mobile health technologies on human papillomavirus vaccination uptake among mothers of unvaccinated girls aged 9–14 years in Lagos, Nigeria (mHealth-HPVac): Study protocol of a randomised controlled trial

**DOI:** 10.21203/rs.3.rs-4541493/v1

**Published:** 2024-06-27

**Authors:** Kehinde S. OKUNADE, Adebola A. ADEJIMI, Temitope V. ADEKANYE, Matthew J. ALLSOP, Hameed ADELABU, Olufemi THOMAS-OGODO, Tonia C. ONYEKA, Teniola LAWANSON, Godwin O. AKABA, Omolola SALAKO, Rose I. ANORLU, Jonathan S. BEREK

**Affiliations:** University of Lagos; University of Lagos; Lagos University Teaching Hospital; University of Leeds; University of Lagos; Lagos University Teaching Hospital; University of Nigeria; University of Lagos; University of Abuja; University of Lagos; University of Lagos; Stanford University School of Medicine

**Keywords:** Cervical cancer, Lagos, mHealth-Cervix, NHIS, Nigeria, Pap test

## Abstract

**Background:**

Despite the availability of effective vaccines, human papillomavirus (HPV) vaccine uptake remains low in most resource-limited settings including Nigeria. Mobile health technology (mHealth) may empower patients to control their health, reduce inequalities, and improve the uptake of HPV vaccination.

**Aim:**

The *“mHealth-HPVac”* study will assess the effects of mHealth using short text messages on the uptake of HPV vaccination among mothers of unvaccinated girls aged 9–14 years and also determine the factors influencing the uptake of HPV vaccination among these mothers.

**Methods:**

This protocol highlights a randomised controlled trial involving women aged 25–65 years who will be enrolled on attendance for routine care at the General Outpatient clinics of Lagos University Teaching Hospital, Lagos, Nigeria between July and December 2024. At baseline, n=224 women will be randomised to either a short text message or usual care (control) arm. The primary outcome is vaccination of the participant’s school-age girl(s) at any time during the 6 months of follow-up. The associations between any two groups of continuous variables will be tested using the independent sample t-test (normal distribution) or the Mann-Whitney U test (skewed data) and that of two groups of categorical variables with Chi-square (*X2*) or Fisher’s exact test where appropriate. Using the multivariable binary logistic regression model, we will examine the effects of all relevant sociodemographic and clinical variables on HPV vaccination uptake among mothers of unvaccinated but vaccine-eligible school-age girls. Statistical significance will be defined as A *P*<0.05.

**Discussion:**

The mHealth-Cervix study will evaluate the impact of mobile technologies on HPV vaccination uptake among mothers of unvaccinated but vaccine-eligible school-age girls in Lagos, Nigeria as a way of contributing to the reduction in the wide disparities in cervical cancer incidence through primary prevention facilitated using health promotion to improve HPV vaccination uptake.

**Registration:**

PACTR202406727470443 (6^th^ June 2024).

## Introduction

In 2020, there were an estimated 604,000 new cases of cervical cancer and 342,000 deaths from cervical cancer, with 70% of these deaths occurring in developing countries [1]. Persistent infection of the cervix with certain high-risk genital human papillomavirus (HPV) is a necessary cause of cervical cancer [2, 3]. One of the most effective strategies for cervical cancer prevention is vaccination against HPV infection among young and adolescent girls before the start of sexual activity [2, 4].

Administering HPV vaccines in low and middle-income countries is essential for achieving the global action plan aimed at bridging the cervical cancer gap [5]. HPV vaccine rollout to girls aged 9–14 years as recommended by the World Health Organization (WHO) [6] is projected to have a greater and faster direct impact and herd effects on the population’s immunity [7]. There are four vaccines against HPV infections[8, 9] and only two of these (quadrivalent Gardasil and bivalent Cervarix) are currently approved for use by the Nigerian Federal Ministry of Health [10, 11]. In October 2023, Nigeria embarked on new routine HPV vaccine roll-out campaigns to reach about 7.7 million girls [12]. Under this new immunization protocol, girls aged 9 to 14 years will receive a single dose of the vaccine [12], which is highly effective at preventing infection of HPV types 16 and 18 that are known to cause at least 70 per cent of cervical cancers [2].

Despite the availability of effective vaccines, HPV vaccine uptake remains low in most resource-limited settings including Nigeria [5, 12, 13]. The utilization of mobile technologies has increased significantly in recent years [14] with increased opportunities for mobile health technologies (mHealth) development [15]. Mobile health technology (mHealth) may empower patients to control their health, reduce inequalities [16], and improve the uptake of health interventions such as HPV vaccination. There are only a few reported studies in Sub-Saharan Africa that examined the use of mHealth in cancer prevention [16] but there are currently none that have investigated the impact of this intervention on the uptake of HPV vaccination among mothers of eligible unvaccinated vaccine-eligible school-age girls.

The primary objective of this proposed study *“mHealth-HPVac”*, therefore, is to assess the effects of mHealth using short text messages on the uptake of HPV vaccination among mothers of unvaccinated girls 9–14 years while the secondary objective is to determine the factors affecting the uptake of HPV vaccination among mothers of unvaccinated girls aged 9–14 years under usual conditions. This is innovative because the introduction of mHealth, now regarded as one of the most promising investments for health in developing countries [17], is a novel concept in the paradigm shift for disease prevention which can contribute to improving cervical cancer control in the resource-limited settings of sub-Saharan Africa (SSA). Thus, the study will generate the first real-world evidence in SSA on the efficacy of mobile technologies on HPV vaccination uptake among eligible school-age daughters of participating mothers in Nigeria.

## Methodology

### Study design and setting

*“mHealth-HPVac”* is a randomised parallel arm controlled trial of mothers of unvaccinated girls aged 9–14 years who attend routine care at the General Outpatient (GOP) clinics of the Lagos University Teaching Hospital (LUTH), Nigeria between June and October 2024. LUTH is the foremost healthcare institution in Lagos and serves as a referral centre for other government-owned and private hospitals in Lagos and its neighbouring states. The hospital provides various care including integrated gynecologic oncology prevention services such as Pap smearing, human papillomavirus testing, colposcopy and pathologic services, including cytology and histology [18]. The GOP clinic of the hospital is opened on each day of the week with attendees being mainly enrollees of the National Health Insurance Scheme.

### Study population

Eligible participants are mothers of unvaccinated girls aged 9–14 years; who express willingness to vaccinate their children; own and use a personal cellphone; free from any mental or physical disabilities that inhibit them from understanding the implications of the study and not considering relocating from their current residence within the next year. The exclusion criteria include refusal of consent or withdrawal of consent during the study.

### Study endpoints and sample size calculation

The *primary endpoint* is the prevalence of single-dose HPV vaccination uptake after 6 months and the *secondary endpoints* are predictors of HPV vaccination uptake after 6 months of enrolment among participating mothers of young or adolescent girls aged 9–14 years. Participants will be tracked via medical record review as well as through phone calls in the 7th month after their enrolment to collect data on their HPV vaccination uptake. With pooled HPV vaccine uptake prevalence of 28.5% for a usual care condition derived from the systematic review by Asgedom et al [19] and an expected attrition rate of 20%, a sample size [20] of n=123 women is expected to provide 80% power to establish a 30% proportional difference in HPV vaccination uptake between mHealth intervention and usual care condition.

### Participants’ enrolment and data collection

**Figure 1:**
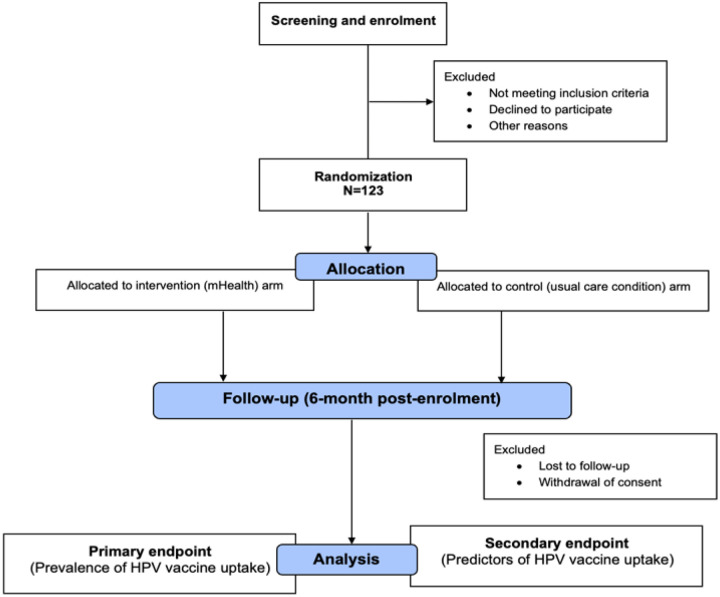
Trial flow chart

A 20–30-minute health talk on cervical cancer prevention including HPV vaccination is given by the clinic midwives as part of usual care to all women in the clinics after which the investigators or research assistants will screen and identify eligible women for the study [Figure 1]. The women will then be invited to give consent for participation upon explanation of the purpose and procedures of the study. Once consent is obtained, an electronic interviewer-administered questionnaire created on the REDCap database will be applied to each participant to obtain baseline information on sociodemographic variables, cellphone use and distance of participants’ residence from the clinics (measured in kilometres using Google map).

### Randomization and allocation concealment

Once enrolled, eligible women will be randomised to either a text message (intervention) arm or a usual care (control) arm using a computer-generated random sequence generated by an independent statistician.

- *Intervention (mHealth) arm* – MultiTexter Bulk short message service (SMS) will be used as the platform to deliver the mHealth messages given its reliability and low cost. Participants will be sent messages containing information on cervical cancer and are then encouraged to take their daughters aged 9–14 years for HPV vaccination. Text messages will be delivered monthly for 6 months after enrollment.
- *Usual care (control) arm* – Participants in this study arm will only receive the usual health education talk at enrolment. They will receive no additional follow-up text messages.

Group allocations will be concealed from the investigators and trial statistician using sealed opaque envelopes that are sequentially numbered. The envelopes will be prepared in advance of the trial and will only be opened sequentially at the time of participants’ enrollment, ensuring that the treatment allocation remains concealed until the point of assignment. Given that no safety concerns are expected, we do not anticipate the need for unblinding during the study.

### Data management and statistical analysis

Data collection will occur prospectively with data entry and checking taking place continuously. Queries will be followed vigorously to ensure clarification without delay. Participants’ enrolment will be facilitated by providing clear, concise, and understandable information about the study’s purpose, procedures, benefits, and risks using compelling language and visuals to make the study interesting and engaging. Each participating woman will be offered free cervical cancer screening if desired and will receive an estimated $2.50 credit charge on their cellphones as a token to enable the investigators to keep their phone numbers for as long as the intervention lasts. We also anticipate some participants’ fatigue and drop-outs during the study’s follow-up period. We will, therefore, mitigate these by training and providing support to the study team on study protocols, including retention techniques such as good communication skills, cultural competence, and specialized knowledge of the target population. The intention-to-treat principle will be used in the final analysis. Data will be analyzed using SPSS version 29.0 for Windows. The participants’ characteristics, by intervention arm, will be presented as mean (standard deviation), median (25th–75th centile), and frequency (%) depending on type and distribution. The significance level is *P*<0.05 and all hypothesis testing will be two-sided. We will test the associations between the intervention and HPV vaccination uptake using Pearson’s Chi-square (χ2) or Fisher’s exact test where appropriate. Using multinomial logistic regression analysis, we will adjust for the effects of all possible covariates in this association. In a subgroup analysis of women in the usual care arm, a multivariable binary logistic regression model will be developed using a backward stepwise selection approach to identify factors such as participant’s age, socioeconomic class, parity, marital status, number of GOP clinic attendance, the distance of residence from the clinics, the functionality of cellphones, adolescent girl’s age and school level and other relevant demographic and clinical variables as that are independently associated with HPV vaccination uptake after 6-months of follow-up. Variables associated with vaccination uptake (*P*<0.10) in the bivariable analyses will be included in the pool of variables for the backward stepwise regression model. An Akaike’s Information Criterion will be generated constantly and the last model step with the smallest AIC is then selected as the best-fit model. Associations in the final model are regarded as significant if *P*<0.05.

### Ethical considerations

Ethical approval for the *“mHealth-HPVac”study* was obtained from the Health Research Ethics Committee of the Lagos University Teaching Hospital (ADM/DSCST/HREC/APP/6566 – May 10, 2024) and the College of Medicine, University of Lagos (CMUL/HREC/5/24/1464 – May 15, 2024). The purpose and nature of the study will be explained to all potential participants and the willing participant will sign an informed consent form. The trial will be reported in line with the Standard Protocol Items: Recommendations for Interventional Trials (SPIRIT) checklist.

### Quality control and data monitoring

All investigators and research assistants will be required to undergo training including good clinical practice (GCP) training before the trial to guarantee consistent practice. The training will include an understanding of inclusion/exclusion criteria, follow-up procedures, and completion of the questionnaire. Identifiable data will be transferred to an electronic database system located in a guarded facility at the trial site by the research assistant. Access to identifiable data is restricted only to the principal investigator (KSO) during and after the trial completion. The trial will be monitored by quality assurance personnel from the research management office of the College of Medicine, University of Lagos, who will be independent of the study team, and an independent steering committee. There will be periodic monitoring to guarantee data accuracy and quality throughout the study period. The essential documents (consent information, enrolment, protocol deviations, and losses to follow-up) will be monitored and checked for accuracy and completeness by the monitors.

### Dissemination of information

The trial will be reported per the Consolidated Standards of Reporting Trials (CONSORT) checklist and the results will be disseminated in a peer-reviewed scientific journal. Important modifications to the trial protocol will be communicated to the funder, study investigators, LUTH and CMUL HREC, trial participants and trial registries.

### Study Status

At the time of manuscript submission, recruitment is yet to commence into the trial. Participants’ enrolment will start in July 2024, and the last woman is expected to be included in the trial in September 2024. The tentative trial completion date is March 2025. This manuscript reports protocol version 4.0 (6th June 2024).

## Discussion

Cervical cancer, the most common HPV-associated malignancy, is a major public health disease in Nigeria. However, despite the availability of effective vaccines, HPV vaccine uptake remains low in most resource-limited settings including Nigeria [5, 12, 13]. The utilization of mobile technologies has increased significantly in recent years[14] with increased opportunities for mobile health technologies (mHealth) development [15]. Mobile health technology (mHealth) may empower patients to control their health, reduce inequalities [16], and improve the uptake of health interventions such as HPV vaccination. Currently, no study has investigated the use of mobile technologies to enhance HPV vaccination uptake. This protocol, therefore, describes a randomised controlled trial of mHealth technologies using text messages to improve HPV vaccination uptake by evaluating the impact of mobile technologies on HPV vaccination uptake among mothers of unvaccinated girls 9–14 years in Lagos, Nigeria and also determine the factors that affect this vaccination uptake as a means of reducing the disparities in the incidence of cervical cancer through primary prevention facilitated using health promotion to improve HPV vaccination uptake. We have powered the study to detect the primary outcome to show that mHealth can improve HPV vaccine uptake in mothers of unvaccinated girls aged 9–14 years. We believe that if found to be effective, the mHealth intervention strategy may become an important tool for reducing the cervical cancer burden, and its associated morbidity and mortality. This approach could also be utilized for health promotion and prevention efforts targeting other significant diseases in the future. However, a limitation of this trial is that we have not specifically powered to detect the predictors of HPV vaccination uptake in these mothers (although this is a planned secondary outcome). The trial would need to be significantly larger to detect these influencing factors. However, the study will generate preliminary data for hypothesis testing in a future robust and carefully designed prospective cohort study.

## Data Availability

No data are associated with this article. The authors intend to grant public access to the full protocol, participant-level dataset, and statistical code associated with this study.
